# Influence of perceived difficulty of cases on student osteopaths’ diagnostic reasoning: a cross sectional study

**DOI:** 10.1186/s12998-017-0161-z

**Published:** 2017-12-01

**Authors:** Aurelien L. Noyer, Jorge E. Esteves, Oliver P. Thomson

**Affiliations:** 1 Research Centre, University College of Osteopathy, 275 Borough High Street, London, UK; 2 Instituto Piaget, VN Gaia School of Health Care, Alameda Jean Piaget, Gulpilhares, 4405-678 Vila Nova de Gaia, Portugal; 3 Clinical-based Human Research Department, Centre for Osteopathic Medicine Collaboration, Pescara, Italy; 4 Osteopathic Health Centre, Dubai, United Arab Emirates

**Keywords:** Cognitive science, Clinical decision-making, Diagnosis, Patient safety, Thinking, Students, Diagnostic errors, Problem-based learning, Intuition, Education

## Abstract

**Background:**

Diagnostic reasoning refers to the cognitive processes by which clinicians formulate diagnoses. Despite the implications for patient safety and professional identity, research on diagnostic reasoning in osteopathy remains largely theoretical. The aim of this study was to investigate the influence of perceived task difficulty on the diagnostic reasoning of students osteopaths.

**Methods:**

Using a single-blinded, cross sectional study design, sixteen final year pre-registration osteopathy students diagnosed two standardized cases under two context conditions (complex versus control). Context difficulty was manipulated via verbal manipulation and case order was randomized and counterbalanced across subjects to ensure that each case was diagnosed evenly under both conditions (i.e. half of the subjects performed either case A or B first). After diagnosis, participants were presented with items (literal, inferred and filler) designed to represent analytical and non-analytical reasoning. Response time and error rate for each item were measured. A repeated measures analysis of variance (concept type x context) was performed to identify differences across conditions and make inferences on diagnostic reasoning.

**Results:**

Participants made significantly more errors when judging literal concepts and took significantly less time to recognize filler concepts in the complex context. No significant difference in ability to judge inferred concepts across contexts was found.

**Conclusions:**

Although speculative and preliminary, our findings suggest the perception of complexity led to an increased reliance on analytical reasoning at the detriment of non-analytical reasoning. To reduce the associated cognitive load, osteopathic educational institutions could consider developing the intuitive diagnostic capabilities of pre-registration students. Postgraduate mentorship opportunities could be considered to enhance the diagnostic reasoning of professional osteopaths, particularly recent graduates. Further research exploring the influence of expertise is required to enhance the validity of this study.

**Electronic supplementary material:**

The online version of this article (10.1186/s12998-017-0161-z) contains supplementary material, which is available to authorized users.

## Background

Diagnostic reasoning (DR) refers to the cognitive processes by which healthcare practitioners formulate diagnoses [1]. Faults in these processes such as cognitive biases have been attributed as a cause of diagnostic error in medicine, thus a wealth of research has been directed at understanding their intricacies [2]. However these abstract concepts are inherently difficult to study as they reside in the clinicians’ mind [1]. Furthermore many of the stipulated recommendations, such as cognitive forcing strategies, lack supporting evidence and thus remain controversial if not difficult to apply [3].

Initiated by Tversky & Kahneman’s [4] early work on the role of heuristics in decision-making, much of the research and theoretical literature on reasoning is based on dual-process theories [5, 6]. Despite focusing on different aspects of the decision-making process, all of these theories share the common principle that reasoning is governed by two distinct cognitive systems [6]. This principle was then applied by Croskerry [7] to generate a pragmatic unifying model describing each system and their interplay during DR.

System one (‘pattern-recognition’ or ‘non-analytical reasoning’) is an unconscious, inductive process by which the recognition of signs, symptoms and other features associated with previously acquired and stored ‘illness scripts’ leads to recall of that particular script and rapid diagnosis formation [8]. Characteristic of 'expert' reasoning, this effortless approach is automatically recruited in pathognomonic scenarios [7]. In contrast, System two (‘hypothetico-deductive’, ‘analytical’ or ‘reflective’ reasoning) is conscious and deductive, relying on the systematic integrative analysis of gathered evidence with pathophysiological mechanisms [1]. Characteristic of novice reasoning, this demanding approach is also solicited in complex situations [7]. Despite its efficiency, the association of System one with cognitive bias and error in unfamiliar scenarios has led to most of the research favouring analytical approaches [2, 9, 10].

Nonetheless, the finding that analytical reasoning may provide a diagnostic advantage for expert practitioners diagnosing complex cases remains questionable [11]. Several authors [11, 12] have attributed this finding to biased experimental designs which interfere with the participant’s cognition. This was supported by Monteiro et al. [13] who demonstrated that when provided with the choice to reflect, very few medical residents did so. Furthermore, additional reflection had no significant effect on diagnostic accuracy.

From these equivocal findings, several sources [12–14] have emphasized the need to understand the moderators that influence the selection of these Systems, such as expertise/experience and task difficulty, rather than establishing the absolute superiority of one over the other. Identifying the circumstances that favour a particular reasoning approach may provide a more flexible alternative to cognitive ‘forcing’ strategies [12]. As a result, clinicians may be more inclined to apply these tactics in practice, perhaps improving diagnostic accuracy and patient care.

In accordance with these suggestions, Mamede et al. [2] successfully employed a decision task to demonstrate the influence of context difficulty in eliciting a shift to analytical reasoning. Contrary to classical diagnostic tasks, this experimental approach adopted from cognitive science researchers [15] is comprised of a diagnostic task followed by a decision task; whereby performance in the latter rather than the former is utilized to make inferences on DR. Instead of relying on the inaccurate ‘time-to-diagnosis’ measure [11], inferences are derived from the ease by which participants can recognize a variety of items designed to represent analytical or non-analytical reasoning [2]. Not only does this novel protocol bypass the aforementioned limitation, but it also provides a means to explore reasoning without the necessity to directly interfere with participant cognition [2].

In the UK, Australia and New Zealand osteopaths are primary healthcare practitioners and are therefore required to possess the ability to diagnose and manage patients presenting with musculoskeletal and non-musculoskeletal complaints [16–18]. Given the repercussions of misdiagnosis on patient care and safety [19], the ability to demonstrate well-developed clinical reasoning skills has been emphasized by these osteopathic regulatory organizations [16–18]. DR constitutes as a part of clinical reasoning, therefore it should be well incorporated into osteopathic practice and education to ensure patient safety and optimal care while upholding the profession’s reputation [1, 20, 21]. Despite these consequences, most of the research on osteopathic DR remains largely theoretical [1].

A recently published qualitative study by Thomson et al. [1] recognized the similarity in DR approach adopted by experienced osteopaths and other healthcare professions including medicine and physiotherapy [19, 22]. Future research on osteopathic DR may therefore benefit from exploiting the recommendations provided by the medical literature, namely the need for ecological designs with a focus on the influence of contextual factors.

The aim of this present study was to replicate Mamede et al.’s [2] experiment to investigate the influence of perceived task complexity on the DR of student osteopaths. Extrapolating from Mamede et al.’s [2] findings, it was hypothesised that perceived task complexity would influence reasoning mode. Specifically, students would rely more heavily on System two compared to System one when presented with a scenario that was perceived to be a complex one. While meeting the need for ecological designs, this investigation may corroborate the findings of Thomson et al. [1], adding to the scarce evidence base supporting osteopathic DR. Exploring the influence of perceived context complexity on reasoning mode may have implications for future research attempting to understand the development of expertise and metacognitive capacities, both of which may guide the formation of educational strategies to enhance patient care [1, 14].

## Methods

The methods are reported in accordance with the CONSORT (2010) statement guidelines [23].

### Design & Setting

A single-blinded, cross-sectional design with two within-subject variables, ‘context’ (control versus complex) and ‘concept type’ (literal, inferred and filler) was employed. The dependent variables were participant mean response time (RT) and mean error rate (ER) per concept type. The study was conducted at the University College of Osteopathy (UCO).

### Participants

#### Recruitment

Sixteen pre-registration osteopathy students volunteered to participate in this study after being informed of the associated risks. The study was advertised via email and written informed consent was obtained from all subjects by the principal researcher. All participants were recruited throughout November and December 2016. This study was approved by the UCO Research & Ethics Committee.

#### Sample size

Assuming an effect size of *f* = 0.3 (estimated from Mamede et al. [2]), power (1-*β*) of 0.8 and a significance level (*α*) of 0.05, sample size was set at 14 subjects (G*Power (3.1.9.2) [24]).

#### Selection criteria

##### Inclusion criteria

All participants were final year 4 pre-registration students on the full-time 4-year Masters of Osteopathy (M.Ost) programme at the UCO. At the time of the study the subjects had completed ~800 h of supervised clinical practice.

##### Exclusion criteria

Participants working in healthcare professions and/or with qualifications pertaining to a recognized healthcare profession acquired prior or during the M.Ost course were excluded.

### Materials & apparatus

The materials consisted of two musculoskeletal clinical cases [see Additional file 1] obtained from a previous study by Esteves [25], which adapted Rikers et al.’s [15] experiment in the context of osteopathic practice.

Akin to Mamede et al. [2], case descriptions included contextual information, presenting complaint, medical history and examination findings. One case (A) described a middle aged woman with lower-back pain and the other (case B) an elderly man with neck pain. Case descriptions were one page long and comprised of 130 (A) and 112 (B) propositions respectively. Both tasks were purposefully complex to promote the recruitment of System two which is unlikely to function in simplistic patient presentations [2]. To ensure familiarity with the procedure, a shorter practice case describing a young man with lower abdominal pain was trialled prior to the experimental scenarios. Following diagnosis, participants were presented with concepts and required to judge their relatedness to the presented case. Forty-eight items per case classified into three concept types were employed: 8 ‘literal’ concepts consisting of direct transpositions of signs and symptoms from the case, 16 ‘filler’ concepts consisting of unrelated signs and symptoms from comparable scenarios and 24 ‘inferred’ concepts. Inferred concepts were extracted from transcriptions of think aloud protocols performed on expert osteopaths [25]. All 24 inferred concepts could be formed on the basis of at least 2 propositions from the scenarios and were divided into 8 clinical, biomedical and osteopathic terms [25].

For instance, the text associated with case B describing a 71-year-old male retiree contained the following information: “…presents with right-sided neck and scapular pain, which started 9 months ago when he hit the ground whilst playing golf. The pain is aggravated by neck movements and relieved by taking Paracetamol and by keeping his neck straight. He reports that although the pain is associated with neck movements, there is increased pain and stiffness on waking, decreasing within 30 minutes…” [25].

*Facet osteoarthritis* would represent a clinical term, *disc degeneration* a biomedical term and *capsular pattern* an osteopathic term. All of these would constitute inferences as they are not explicitly stated in the text but supported by the information provided. In accordance with the aforementioned definitions, *scapular pain* would represent a literal concept and *frontal headache* a filler concept. All materials were presented on a HP Presario CQ27 laptop computer using the SuperLab 4.0.3 software (Cedrus Corp., San Pedro, California). Subjects provided their diagnosis(es) electronically via the laptop keyboard. An 8-button Cedrus Response Box (RC-830, Cedrus Corp., San Pedro, California) was employed for the decision task. Participants were instructed to press the green key for related items and the red key for unrelated items. To maximize accuracy all subjects maintained each index finger on the corresponding key (left on red, right on green) [25].

### Procedure

#### Overview

The procedure followed the main layout provided by Mamede et al. [2] with some minor amendments obtained from Esteves [25] to accommodate for the osteopathic case scenarios. The latter were longer and contained more concepts than that employed by Mamede et al. [2]. The experiment consisted of a practice case followed by 2 consecutive testing blocks, each corresponding to either case A or B. Each block was composed of three sequential trials: the case description and diagnosis, the instructions for the decision task and lastly the consecutive presentation of items.

All participants were tested individually in the same room located in the UCO teaching facility. Each session lasted ~30 min and subjects were allowed to rest ad libitum in between blocks.

#### Intervention, randomization & blinding

Participants were instructed to provide the best fitting diagnosis(es) for the case description prior to each block. No additional information was provided for the first case, however when presented with the second, participants were informed that experienced osteopaths had failed to diagnose it correctly. This verbal manipulation was successfully employed by Mamede et al. [2] to induce a perception of complexity.

Although the manipulation was provided prior to the second block, case order was counterbalanced across subjects to ensure that each case was diagnosed evenly under both conditions (i.e. half of the subjects performed either case A or B first). Item order was randomized (simple randomization) by means of a computer-generated sequence and participants were blinded to the manipulation. All randomization and counterbalancing procedures were performed by the principal researcher.

#### Protocol

Participants were instructed to carefully read and study each case description presented on a computer screen for 4 minutes. They were then asked to provide the most likely diagnosis(es) into an entry box by means of the laptop keyboard. This task was not time restricted and subjects pressed ‘enter’ to validate their entry.

Following this, a new screen containing the instructions for the decision task appeared. These remained visible for 10 seconds and instructed the participants to judge whether the presented concepts were related or unrelated to the case by means of the aforementioned response box. Emphasis on speed and accuracy was made to control for the possible confounding effect of further deliberation occurring throughout this task [2]. Prior to the experimental trial, participants were informed that items were considered related if they were (i) literally stated in the case scenarios or (ii) inferences based on pathophysiological/osteopathic mechanisms concluded from information provided in the case description [25]. Participants were not aware of the presence of filler concepts as these were simply unrelated signs and symptoms. Once the time elapsed the instructions were replaced by a fixation cross placed in the middle of the screen for 500 milliseconds. The fixation cross was then substituted by an item marking the beginning of the decision task. All concepts remained visible until a response was made, subsequently, the fixation cross reappeared for the same duration of time. All forty-eight items were therefore interspaced by the 500-millisecond fixation cross. RT and error count for each item was automatically registered by the SuperLab software for both experimental blocks [25].

### Statistical analysis

Data normality was assessed by appraisal of histogram plots, skewness/kurtosis and Shapiro-Wilk tests. RT data were positively skewed thus all analyses were performed on Log-normal transformed values [26]. Despite a statistically significant Shapiro Wilk test (*p* < 0.05), ER data were treated as normal due to the absence of skew and a relatively normal histogram plot.

Mean RT (milliseconds) and mean ER (%) was determined for each concept type under each context. Data were analysed via a two-way repeated-measures analysis of variance (ANOVA) with context and concept type as within-subject factors. Harwell et al. [27] have suggested ANOVAs are robust to moderate normality violations. Effect size (*η*_*p*_^*2*^) for the main and interaction effects were calculated. Post-hoc paired t-tests were used for comparisons across conditions. Significance level was set at *p* ≤ 0.05 for the repeated-measures ANOVA and a Bonferroni-adjusted value of *p* ≤ 0.0055 for the post-hoc analyses. Data was analysed with the JASP (version 0.8.0.0) software [28].

## Results

### Error rate

Table 1 presents the mean ER of the subjects as a function of concept type and context. There was a significant main effect of context, *F*(1, 15) = 11.87, *p* = 0.004, *η*_*p*_^*2*^ = 0.442, and concept type, *F*(2, 30) = 5.37, *p* = 0.01, *η*_*p*_^*2*^ = 0.264. The interaction between context and concept type was also significant, *F*(2, 30) = 7.29, *p* = 0.003, *η*_*p*_^*2*^ = 0.327.

**Table 1:** Mean error rate (%) and standard deviation as a function of concept type and context (*N* = 16)

	Control context	Complex context
Concept type	Mean (*SD*)	Mean (*SD*)
Literal	22.7 (17.8)	47.7 (12.3)
Inferred	35.4 (15)	40.6 (15.4)
Filler	43.8 (13.9)	46.9 (10.7)

**Fig. 1:**
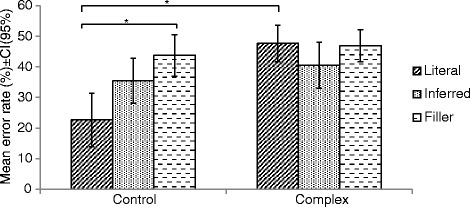
Mean error rates as a function of concept type and context (* = *p* ≤ 0 .0055)

Post-hoc Bonferroni-adjusted t-tests showed that subjects made significantly more errors when judging literal concepts in the complex context (*M* = 47.7, *SD* = 12.3) compared to the control context (*M* = 22.7, *SD* = 17.8), *t*(15) = − 4.57, *p* < 0.001 (Fig. 1). There was no significant difference in ER between contexts for inferred (*p* = 0.30) and filler (*p* = 0.46) concepts. Participants made significantly fewer errors when judging literal concepts (*M* = 22.7, *SD* = 17.8) compared to filler concepts (*M* = 43.8, *SD* = 13.9) in the control context, *t*(15) = − 3.70, *p* = 0.002. There was no significant difference in ER between inferred and literal concepts (*p* = 0.02) or between inferred and filler concepts (*p* = 0.08) in the control context. There was no significant difference in ER across concept types in the complex context (*p* > 0.0055).

### Response time

Table 2 presents the mean RT of the subjects as a function of concept type and context. The main effect of context was not significant, *F*(1, 15) = 1.93, *p* = 0.18, *η*_*p*_^*2*^ = 0.114. Mauchly’s test indicated that the assumption of sphericity for the main effect of concept type was violated (*p* = 0.008), thus degrees of freedom were adjusted using.

**Table 2:** Mean response time (milliseconds) and standard deviation as a function of concept type and context (N = 16)

	Control context	Complex context
Concept type	Mean (*SD*)	Mean (*SD*)
Literal	1922 (711)	2157 (801)
Inferred	2113 (765)	1886 (671)
Filler	2541 (935)	2119 (680)

Greehouse-Geisser estimates of sphericity (*ε* = 0.67). This main effect was significant, *F*(1.3, 20) = 7.85, *p* = 0.007, *η*_*p*_^*2*^ = 0.344. The interaction between context and concept type was significant, *F*(2, 30) = 8.54, *p* = 0.001, *η*_*p*_^*2*^ = 0.363.

**Fig. 2:**
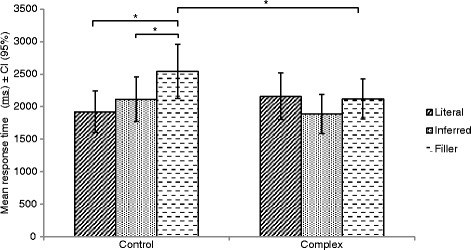
Mean response time as a function of concept type and context (* = *p* ≤ 0.0055)

Post-hoc Bonferroni-adjusted t-tests showed that subjects took significantly less time to judge filler concepts when placed in the complex context (*M* = 2119, *SD* = 680) compared to the control context (*M* = 2541, *SD* = 935), *t*(15) = 3.29, *p* = 0.005 (Fig. 2). There was no significant difference in RT between contexts for inferred (*p* = 0.06) and literal (*p* = 0.13) concepts. Participants judged filler concepts (*M* = 2541, *SD* = 935) significantly slower than inferred (*M* = 2113, *SD* = 765), *t*(15) = − 4.45, *p* < 0.001, and literal concepts (*M* = 1922, *SD* = 711), *t*(15) = − 3.87, *p* = 0.002, in the control context. There was no significant difference in RT between literal and inferred concepts in the control context (*p* = 0.22). There was no significant difference in RT across concept type in the complex context (*p* > 0.0055).

## Discussion

This study investigated the influence of perceived task complexity on the DR of student osteopaths. Akin to previous research [2, 7, 29] it was hypothesized that the perception of complexity would elicit a shift in reasoning mode from System one to two. Greater reliance on the latter would enhance the consideration of alternative hypotheses, thus the quantity of inferences available in the working memory prior to the decision task [2]. Consequently, subjects in the complex context would make fewer errors and respond faster to inferred concepts compared to their control counterparts.

The absence of a significant change in ER and RT for inferred concepts across context does not support our initial prediction. However, this lack of change may be attributed to the limited experience of the sample rather than the absence of a reasoning shift per se.

Unlike experts, novices do not possess an encapsulated knowledge base and are therefore predominantly reliant on analytical processes [15, 25, 30]. The recruited participants, unlike novices, had amassed considerable hours of practice and were deemed to be ‘intermediates’ [15, 25]. Although the latter have extensive biomedical knowledge, it is still vastly pre-encapsulated as clinical knowledge is still under development [15, 25]. Consequently, given the high complexity of the cases, it is possible that subjects were reliant on System 2 throughout both scenarios to an extent that would mask the manipulation effect. In other words, a reasoning shift may have occurred but it was too subtle to appreciate. This hypothesis is supported by the 50% increase in ER for literal concepts in the complex context. Although cognitive overload [29] may account for this observation, it was controlled for by the provision of rest between blocks and the counterbalancing of case order. Furthermore, as Croskerry [7] highlights, one would expect an increased reliance on System one at the expense of System two, thus an improvement in the judgement of literal concepts rather than a detriment. Likewise, the ability to judge inferred concepts would be hindered yet it was unaffected.

This misjudgement of literal concepts may be interpreted as the ‘rational override’ [7] of System one during the diagnostic stage, hindering the capacity of participants to recall repeated signs and symptoms in the subsequent decision task. This is further supported by the reduction in RT for filler concepts which suggests participants, perhaps due to further deliberation, were able to exclude with greater ease similar but unrelated concepts [25].

These findings, although implied and therefore of putative nature, suggest the possibility that the perception of case complexity may have a role in eliciting a shift to analytical reasoning in student osteopaths. This is consistent with the findings of both quantitative [2] and qualitative [1] research into medical and osteopathic DR. The manipulation may have triggered further deliberation by inducing the sensation of uncertainty described by the experienced osteopaths that participated in the qualitative study by Thomson et al. [1]. The hypothesised predominantly analytical approach adopted by the recruited sample in this study is comparable to that of novices described in both medical [7, 29–31] and physiotherapy [19, 22, 32, 33] expertise literature and has implications for the osteopathic profession in the following ways:

Firstly, the focus of osteopathic educational institutions should be that of developing the traits of expertise in students, specifically, a highly organized knowledge base with well-developed intuitive and metacognitive capacities [1, 19, 22, 32]. Fostering the formation of cognitively flexible practitioners, able to maintain an awareness of the influence of context on their thought process, may mitigate the potential diagnostic error associated with System two overreliance [3, 29]. This may be facilitated by early exposure to clinical environments [34] and the inclusion of case-based learning approaches that promote the acquisition of clinically relevant patterns [8, 35, 36]. In combination with a basic understanding of dual-process theory, students should be encouraged to reflect upon their reasoning to refine their illness scripts and reinforce the encapsulation of biomedical and clinical knowledge [3, 11, 32, 33]. Encouragement of this reflective process by tutors during clinic placements may develop the skills (e.g. criticality and independence) associated with evidence informed and patient centred practice [19, 34].

Secondly, expertise is not a mere function of clinical experience [22, 37, 38] and it is important to consider that the recruited sample, which displayed the characteristics of intermediate clinicians, was in the final stage of the course. This raises the issue that professional osteopaths, particularly recent graduates, may benefit from mentorship opportunities following graduation. As Petty & Morley [38] have identified, the plethora of skill-focused courses designed to satisfy continuing professional development (CPD) schemes set by governing organizations may be of limited value in promoting the development of expertise [34]. As an alternative, practitioners of differing experience levels could partake in mutual observation and reflection [38]. This may provide a cost-effective option, beneficial to osteopaths of all experience level in enhancing their practice and should be considered by osteopathic governing organizations.

Despite these implications the study has several limitations which require acknowledgement. Firstly, the absence of a think aloud protocol does not exclude the possibility that events other than the hypothesized reasoning shifts may have been responsible for the observed change in RT and ER [2]. During these protocols participants are required to ‘think aloud’ whilst diagnosing the cases under both conditions; the differences in propositions (inferred and literal) are then employed to support the hypothesized reasoning shifts [2]. Secondly, although participants subjectively reported a sense of uneasiness, there was no objective measure to reliably confirm the effect of the manipulation. Thirdly, unlike that proposed by Mamede et al. [2], ER and RT were never affected concurrently; therefore, further weakening the inferences extrapolated from the decision task. However, this may have occurred due to the masking of the manipulation effect associated with the use of an intermediate rather than expert sample. Fourthly, although a power analysis was performed, the calculation was based from estimates of main effects rather than the interaction effect, thus it is possible that the study was underpowered. Lastly, despite the attempt to provide a less invasive protocol to explore reasoning, the experiment remains largely artificial and the small sample and effect sizes limit the generalizability of the results [2]. Further studies that address these limitations are being conducted.

## Conclusion

While acknowledging their speculative nature, our findings suggest final year student osteopaths are predominantly reliant on analytical approaches. When manipulated to perceive a case as complex, this reliance is enhanced at the detriment of non-analytical reasoning.

To reduce cognitive overload and the potential diagnostic error associated with it, osteopathic educational institutions could consider adopting strategies that nurture the various components of expert DR such as contextualized learning. Osteopathic statutory regulating bodies, health authorities and professional associations could consider the inclusion of postgraduate mentorship programs to enhance the DR of both experienced and novice osteopaths.

Further research employing verbal protocols and larger samples are required to elucidate our tentative conclusions. Mixed methods comparing expert and novice practitioners may provide insight into the influence of context as a function of expertise.

## Additional file

Additional file 1: Musculoskeletal clinical cases A and B. (DOCX 19 kb)

## Abbreviations

ANOVA: analysis of variance
DR: Diagnostic reasoning
ER: Error rate
RT: Response time
UCO: University College of Osteopathy
