# Supplementary material for: Influence of perceived difficulty of cases on student osteopaths’ diagnostic reasoning: a cross sectional study
Source: Chiropr Man Therap. 2017 Dec 1;25:32. doi: 10.1186/s12998-017-0161-z (PMC5709833; doi:10.1186/s12998-017-0161-z)
Supplement: Additional file 1: — Musculoskeletal clinical cases A and B. (DOCX 19 kb) [file 12998_2017_161_MOESM1_ESM.docx]

**Case A scenario**

**Presenting complaint:** A 40-year-old female university lecturer, recently divorced, mother of 2, presents with right-sided low back pain, which started 2 weeks ago after playing golf. In the last week she developed a sharp pain radiating down the back of her right thigh to the outside of her ankle. Sitting, walking and turning in bed aggravate her symptoms. Sleeping on her left side and ibuprofen relieve the pain. She suffers from occasional generalised pelvic discomfort and a ‘weak’ bladder. In addition, she reports increased pain and stiffness on waking, decreasing slightly within an hour as well as bloating, low back and abdominal pain with her menses.

**Past medical history:** Medial collateral ligament sprain of her right knee at the age of 19, playing netball. RTA at the age of 25; right-sided impact, suffered neck whiplash. Low back and symphysis pubis pain during her 2nd pregnancy, 5 years ago, had forceps delivery. Fibroids diagnosed 3 years ago.

**Family history:** Mother has just recovered from breast cancer. Other negative.

**General examination:** She looks tired and has difficulty in maintaining eye contact.

Moderate antalgic gait with a right-sided weight bearing avoidance. Hyperlordotic lumbar and cervical spines. Right-sided lumbar paraspinal and gluteal muscle spasm; tenderness over L5/S1 and right SIJ; oedema over right SIJ; Hypertonicity, tenderness and increased skin moisture of thoraco-lumbar paraspinal musculature. Forward flexion and bilateral side bending limited with pain in the lumbar spine and right SIJ. Squatting causes pain in the right SIJ.

**Specific mobility testing:** Marked restriction to movement at the thoraco-lumbar

junction. Restriction in movement at the left SIJ and at the levels of C1/2/3; C7/T1; T4/5/6;

T9/10 and L3/4. Hypermobility at L5/S1, right SIJ and right knee on medial gapping.

**Pain provocation/special tests:** Increased pain in the right SIJ with compression,

distraction and hip abduction at 90 degrees. Pain on palpation of the right SIJ. Straight leg raising test (SLR) is positive at 60 degrees on the right.

**Neurological:** Normal deep tendon reflexes; no sensory loss; power normal.

**Abdomen:** Suprapubic and right iliac fossa tenderness but no defence.

**List of concepts for Case A:**

**Clinical items (inferred concepts)**

· Acuteness

· Radiculopathy

· T12 Somatic dysfunction

· Sacroiliac dysfunction

· Spondylolisthesis

· Stasis

· Uterine leiomyoma

· Prolapsed intervertebral disc

**Biomedical Items (inferred concepts)**

· Increased sympathetic outflow

· Sacroiliac ligament inflammation

· Capsular inflammation

· Ligament laxity

· Nerve root impingement

· Pelvic floor fibrosis

· Suppressed immune system

· Fibroblast activity

**Osteopathic Items (inferred concepts)**

· Muscle chain problem

· Pelvic-sacral torsion

· Decompensation

· Compensatory pattern

· Viscerosomatic reflex

· Anteriorised innominate

· Facilitated segment

· Littlejohn model

**Signs and symptoms (literal concepts)**

· Radiating pain

· Normal deep tendon reflexes

· Hyperlordotic cervical spine

· Oedema

· Gluteal spasm

· Morning stiffness

· Bloating

· Antalgic posture

**Other signs and symptoms (filler concepts)**

· Paraesthesia

· Anterior pelvic tilt

· Decreased thoracic kyphosis

· Contractured adductors

· Pubic symphysis hypermobility

· Tender sacrotuberous ligament

· Weak gluteus medius

· Weak transversus abdominis

· Sway back posture

· Restricted hip flexion

· Pain when standing

· Night pain

· Posterolateral thigh pain

· Coccygeal restriction

· Anxiety

· Diaphragm hypertonicity

**Case B Scenario**

**Presenting complaint:** A 71-year-old retired man, who plays golf and enjoys gardening, presents with right-sided neck and scapular pain, which started 9 months ago when he hit the ground whilst playing golf. The pain is aggravated by neck movements and relieved by taking Paracetamol and by keeping his neck straight. He reports that although the pain is associated with neck movements, there is increased pain and stiffness on waking, decreasing within 30 minutes. Although he has been able to continue playing golf, he needs to take Paracetamol to ease his neck pain before he starts. He reports that his general health is reasonably good. He needs to go to the toilet at least twice a night but on his last prostate check-up, 4 months ago, apart from a slight prostate enlargement, nothing abnormal was detected.

**Past medical history:** Left hip replacement, 15 years ago. Suffered mini-stroke, 7 months ago. Other negative.

**Family history:** Mother died of stroke in her eighties. Father died of pneumonia, aged 85. Other negative.

**Medication:** Paracetamol, Aspirin and Simvastatin. Other negative.

**General examination:** Hyperlordotic cervical spine. Reduced thoracic kyphosis with a ‘s’ scoliosis; concave right in the thoracic spine and concave left in the lumbar spine. Marked hypertonicity and tenderness of his right trapezius and left scalenes and sternocleidomastoid muscle groups. Hypertonicity of the bilateral scapulothoracic muscle groups, being more marked on the right. Mild tenderness on superficial palpation of the postural interscapular musculature. Tenderness on deep segmental palpation of the erector spinae muscle groups, especially in the cervical and upper thoracic spine. Oedema over CDJ. Marked restriction in active rotation and side bending of his cervical spine. Right sided rotation and side bending of the cervical spine exacerbate his symptoms.

**Specific mobility testing:** Marked restriction to movement at the cervical spine,

particularly at the levels of C0/C1/C2 and C5/6. Restriction to movement at C7/T1, being more marked in right rotation than left, with pain being precipitated on mobility testing. Forward flexion precipitate pain in the interscapular musculature and the origin of the right levator scapulae musculature. Restriction in movement at the left sacroiliac joint and at the vertebral levels of L4/L5/S1. General limitation in movement of the right glenohumeral and scapulothoracic joints.

**Pain provocation/special tests:** Increased pain with compression, right side bending and extension of his cervical spine. Other negative.

**Neurological:** Normal deep tendon reflexes; no sensory loss; power normal. Other

negative.

**CVS:** Blood pressure 148/92 mmHg. Other negative.

**List of concepts Case B**

**Clinical items (inferred concepts)**

· Facet osteoarthritis

· Spondylosis

· C7 Somatic dysfunction

· Transient ischaemic attack

· C1 Somatic dysfunction

· Benign prostatic hyperplasia

· Prolapsed intervertebral disc

· Hyperlipidaemia

**Biomedical Items (inferred concepts)**

· Posterior vertebral osteophytes

· Disc degeneration

· Synovial inflammation

· Rectus capitis hypertrophy

· Subchondral sclerosis

· Atheroma

· Bladder musculature hypertrophy

· Capsular fibrosis

**Osteopathic Items (inferred concepts)**

· Protracted head

· Superior cervical ganglia

· Muscle imbalance

· Capsular pattern

· Pelvic torsion

· Second-degree lesion

· Inter-arch pivots

· Decompensation

**Signs and symptoms (literal concepts)**

· Nocturia

· Scoliosis

· Reduced kyphosis

· Power normal

· Trapezius hypertonicity

· Diastolic 92 mmHg

· Scapular pain

· Restricted C5/6

**Other signs and symptoms (filler concepts)**

· Vertebrobasilar insufficiency

· Dull ache

· Radiating pain

· Joint instability

· Hyperlordotic lumbar spine

· Protracted shoulders

· Frontal headache

· Restricted upper ribs

· Tightness

· Shortened pectorals

· Weak rhomboids

· Contractured psoas

· Numbness

· Postvoid dribbling

· Hypertonic diaphragm

· Weak neck flexors

**Practice case scenario**

A 24-year-old male student, who is a competitive 110-meter hurdler presents with left-sided groin and lower abdominal pain that started six months ago. The problem came on gradually at the beginning of the indoor season and although it did initially improve with some physiotherapy it has since been on and off and it is now becoming impossible for him to continue training without pain. The problem is particularly bad after a hard training session and the pain in the groin and abdominal region can be excruciating when he has to sneeze or cough. He also recently noticed that his left sacroiliac joint and lumbar spine are particularly painful, preventing him from having a good night sleep. Although it is a sensitive subject for him to talk about, he feels that he has recently noticed a decrease in the frequency of his sexual activity due to the pain that he experiences during intercourse.

**List of concepts**

· Radiating pain

· Chronic recurrent

· Inguinal hernia

· Symphysis pubis dysfunction

· Low back pain

· Ankylosing spondylitis

· Night pain

· SIJ inflammation
